# Anticonvulsant, Anxiolytic, and Sedative Activities of *Verbena officinalis*

**DOI:** 10.3389/fphar.2016.00499

**Published:** 2016-12-21

**Authors:** Abdul Waheed Khan, Arif-ullah Khan, Touqeer Ahmed

**Affiliations:** ^1^Department of Pharmacology, Riphah Institute of Pharmaceutical Sciences, Riphah International UniversityIslamabad, Pakistan; ^2^Neurobiology Laboratory, Atta-ur-Rahman School of Applied Biosciences, National University of Sciences and TechnologyIslamabad, Pakistan

**Keywords:** *Verbena officinalis*, anticonvulsant, anxiolytic, sedative, PTZ, EPM

## Abstract

We describe different neuropharmacological effects of *Verbena officinalis* crude extract (Vo.Cr). Pentylenetetrazole (PTZ)-induced seizures, elevated plus maze, light–dark box (LDB), open field and thiopental-induced sleeping test models were employed to evaluate Vo.Cr actions in mice. Vo.Cr dose-dependently (100–500 mg/Kg) delayed onset time of myoclonic jerks and tonic-clonic seizures, while decreased duration of tonic-clonic seizures (*P* < 0.05, *P* < 0.001 vs. saline group). Vo.Cr at 100 and 300–500 mg/Kg doses reduced animals’ mortality in PTZ-induced seizures test to 75 and 0%, respectively. Vo.Cr (50–300 mg/Kg) significantly increased time spent and number of entries into open arms, while decreased time spent and number of entries into closed arms (*P* < 0.05, *P* < 0.01, *P* < 0.001 vs. saline group), measured in elevated plus maze. Vo.Cr (50–300 mg/Kg) increased time spent in light compartment, while decreased time spent in dark compartment (*P* < 0.01, *P* < 0.001 vs. saline group) in LDB, like caused by diazepam. In open field test, Vo.Cr decreased number of ambulations and rearings frequencies, while increased the number of central squares crossings. In thiopental-induced sleeping test, Vo.Cr (50–300 mg/Kg) decreased onset time of sleep, while increased the duration of sleep (*P* < 0.05, *P* < 0.01, *P* < 0.001 vs. saline group). These results indicate that *Verbena officinalis* possess anticonvulsant, anxiolytic and sedative activities, which provides scientific background for its medicinal application in various neurological ailments, such as epilepsy, anxiety, and insomnia.

## Introduction

*Verbena officinalis* is a perennial herb, belongs to family Verbenaceae, commonly known as “Vervian,” “Herb of grace,” “Pigeon’s grass” and localy “Karenta” or “Pamukh.” It mostly grows in Europe and Asia, commonly found in cultivated fields and wastelands near water and cultivated in Northern and Western areas of Pakistan (Jafri et al., 1974; Khan et al., 2015). It grows up to height of about 1 m, having lobed and toothed leaves, while flowers are elegant, silky and pale purple in color (Voogelbreinde, 2009). In traditional system of medicine, *Verbena officinalis* has been used for treatment of melancholia, hysteria, seizures, jaundice, fever, cholecystaliga, anxiety, depression, insomnia, menstrual disorders (Khare, 2007), abdominal problems, malaria, pharyngitis, edema (Kou et al., 2013), cough, asthma (Vitalini et al., 2009), rheumatic and thyroid problems (Guarrera et al., 2005) etc. The constituents isolated from *Verbena officinalis* include verbenin, verbenalin, hastatoside, alpha-sitosterol, ursolic acid, oleanolic acid (Duke, 1992), kaempferol, luteolin (Chen et al., 2006), verbascoside, aucubin, apigenin, scutellarein (Rehecho et al., 2011) and essential oils like limonene, cineole, spathulenol, ar-curcumeme (Chalchat and Garry, 1995). The plant is reported to possess antitussive (Gui and Tang, 1985), analgesic, anti-inflammatory (Calvo, 2006), neuroprotective (Lai et al., 2006), Antiradical (Speroni et al., 2007), antioxidant, antifungal (López et al., 2008), anti-tumor (Kou et al., 2013), antibacterial (Mengiste et al., 2015), Antiproliferative (Encalada et al., 2015), and antidepressant (Kamal et al., 2015) activities. In this investigation, we report anticonvulsant, anxiolytic and sedative actions of *Verbena officinalis*, which explains its ethno-medicinal use in neurological disorders.

## Materials and Methods

### Plant Material and Extraction

*Verbena officinalis* whole plant (3.2 Kg) was collected from National Industrial State, Rawat, Islamabad, Pakistan and was verified by Dr. Mushtaq Ahmad, a Plant Taxonomist, Department of Plant Science, Quaid-e-Azam University, Islamabad, Pakistan. A voucher specimen number 270 was deposited at the same Department for future reference. The plant material was shade dried until water evaporated completely and then grinded. The coarse powder (2.7 Kg) was macerated in 70% aqueous-methanol under room conditions for 2-weeks with gentle occasional shaking. The extract was filtered and then concentrated in vacuum under reduced pressure using rotary evaporator to obtain a blackish thick paste of *Verbena officinalis* crude extract (Vo.Cr), which was completely solublized in normal saline (0.9% w/v). The percentage yield of extract was found to be 18.66% w/w.

### Animals

Swiss albino mice (25–35 g) of either sex were used for this study, housed in Animal House of Riphah Institute of Pharmaceutical Sciences in polypropylene (22 cm × 37 cm) cages under standard laboratory environment; 25 ± 2°C, light and darkness duration were for 12 h each and had free access to standard diet and water *ad libitum*. Experiments performed complied with rules of Institute of Laboratory Animal Resources, Commission on Life Sciences University, National Research Council (1996), approved by Ethical Committee of Riphah Institute of Pharmaceutical Sciences (Ref. No: REC/RIPS/2015/006).

### Drugs and Chemicals

Diazepam (Valium 10 mg/2 mL injection), pentylenetetrazole (PTZ) and thiopental sodium (Pentothal 500 mg dry powder for injection) were obtained from Roche Pharmaceuticals, Pakistan, Sigma-Aldrich, Co. LLC, USA and Abbot Laboratories, Pakistan, respectively.

## Experimental Protocols

### Anticonvulsant Study

#### PTZ-Induced Seizures

Mice were divided into five groups (each having four animals) and injected (i.p) with normal saline (10 mL/Kg), Vo.Cr (100–500 mg/Kg), and diazepam (1 mg/Kg). Thirty min later to saline, Vo.Cr and diazepam treatment, an i.p. dose of PTZ (90 mg/Kg) was given to all animals and each animal was observed for onset time of myoclonic jerks and tonic-clonic seizures, as well as duration of tonic-clonic seizures for 30 min. Drugs that delayed onset of myoclonic jerks, tonic-clonic seizures and/or shortened duration of tonic-clonic seizures are considered to exhibit anticonvulsant effect (Bum et al., 2008; Ya’u et al., 2008). The animals were also observed for mortality (% mortality = number of mice died after convulsion/total number of mice used × 100).

### Anxiolytic Assays

#### Elevated Plus Maze (EPM)

Test EPM model consist of a wooden apparatus having four arms (two open arms of 30 cm × 5 cm and two closed arms of 30 cm × 5 cm × 15 cm) connected via a central opened area (5 cm × 5 cm), elevated up to 40 cm from the floor, as previously described (Karim et al., 2012). The edges of open arms are surrounded by 3 mm high and 1 mm thick wall. Mice were divided into five groups (each having four animals), received an i.p. dose of normal saline (10 mL/Kg) and Vo.Cr (50–300 mg/Kg) once/day for 7 days, while one group was treated with diazepam (2 mg/Kg), injected once time 30 min prior to test. After 30 min of saline, Vo.Cr and diazepam treatment, a 5 min test was performed by placing each mouse on central area and recorded time spent and number of entries into open and closed arms, via a digital video camera. An entry was considered, when mouse placed all four paws in any arm. The total number of entries into and total time spent in open and closed arms were calculated for determining anxiolytic activity. Following each trial, apparatus was cleaned with 70% aqueous-ethanol.

#### Light–Dark Box (LDB) Technique

Light–dark box (LDB) model contains wooden box of dimensions 44 cm × 21 cm × 21 cm and divided into a small compartment (1/3rd), painted black inside and covered with a wooden lid and a large compartment (2/3rd) which was painted white, covered by a transparent glass and illuminated by a 60 watt bulb. The two compartments were separated by a wooden blank, having a hole of 7 cm × 7 cm in center at surface of floor. Mice were divided into five groups (each having four animals), received an i.p. dose of normal saline (10 mL/Kg) and Vo.Cr (50–300 mg/Kg) once/day for 7 days, while one group was treated with diazepam (2 mg/Kg), injected once time 30 min prior to test. After 30 min of saline, Vo.Cr and diazepam treatment, a 5 min test was performed by placing each mouse in center of lighted box, keeping face away of opening hole and time spent in each compartment was recorded via a digital video camera (Belzung et al., 1987; Bourin and Hascoet, 2003).

#### Open Field Test

The apparatus is made of a wooden base divided into 12 equal squares with glass walls of dimensions (50 cm × 25 cm × 50 cm). Mice were divided into five groups (each having four animals), received an i.p. dose of normal saline (10 mL/Kg) and Vo.Cr (50–300 mg/Kg) once/day for 7 days, while one group was treated with diazepam (2 mg/Kg), injected once time 30 min prior to test. After 30 min of saline, Vo.Cr and diazepam treatment, a 5 min test was performed by placing each mouse in corner square to explore the arena and recorded numbers of ambulations, rearings, and central squares crossings (Brown et al., 1999; Maurmann et al., 2011), via digital video camera. Ambulations mean number of total squares crossed by mice, rearings are the number of times mice stood on its hind limbs and central squares crossings are the number of times mice entered the central squares with its all four paws. Central squares are those that are not adjacent to arena walls. A square number was counted when mice enter in square with all four paws.

### Sedative Activity

#### Thiopental-Induced Sleeping Assay

Thiopental-induced sleeping test was used for determining sedative activity (Alnamer et al., 2012). Mice were divided into five groups (each having four animals), received an i.p. dose of normal saline (10 mL/Kg), Vo.Cr (50–300 mg/Kg), and diazepam (3 mg/Kg). Thirty min later to saline, Vo.Cr and diazepam treatment, an i.p. dose of thiopental sodium (50 mg/Kg) was given to all animals for induction of sleep. As thiopental sodium was injected, mice were placed in a test arena for observing the onset and duration of sleep.

#### Acute Toxicity Test

Mice in two groups were administered with Vo.Cr high doses of 3 and 5 g/Kg, i.p, then kept under observation for 24 h and observed for toxicity symptoms or death.

### Statistical Analysis

Data expressed are mean ± standard error of mean (SEM, *n* = number of experiments). The statistical parameter applied is one-way analysis of variance (ANOVA) with Tukey *post hoc* test. *P* < 0.05 was noted as significantly different. The bar-graphs were analyzed using Graph Pad program (GraphPAD, San Diego, CA, USA).

## Results

### Effect on PTZ-Induced Seizures

**FIGURE 1:**
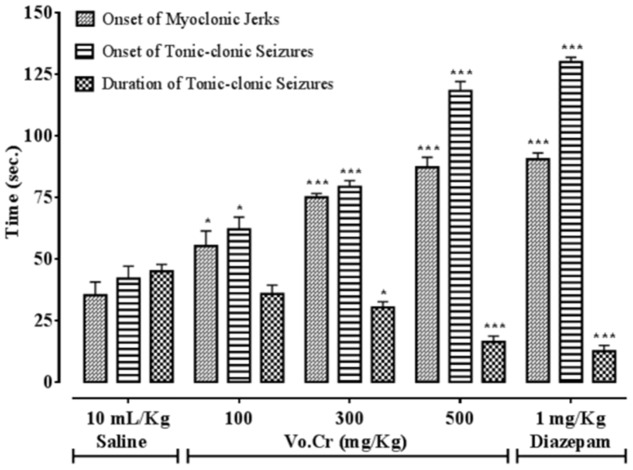
**Bar-graph showing effects of *Verbena officinalis* crude extract (Vo.Cr) and diazepam on onset time of pentylenetetrazole-induced myoclonic jerks, tonic-clonic seizures and duration of tonic-clonic seizures in mice.** Data expressed as mean ± SEM, *n* = 4. ^∗^*P* < 0.05, ^∗∗∗^*P* < 0.001 vs. saline group, one-way ANOVA with *post hoc* Tukey test.

Vo.Cr dose-dependently (100–500 mg/Kg) delayed onset time of PTZ (90 mg/Kg) mediated both myclonic jerks and tonic-clonic seizures, while decreased duration time of tonic-clonic seizures (**Figure 1**). In control saline group, onset times of myoclonic jerks, tonic-clonic seizures and duration of tonic-clonic seizures were 35.25 ± 5.39, 42 ± 5.05, and 45 ± 2.74 s, respectively. In Vo.Cr (100 mg/Kg) treated group, onset times of myoclonic jerks and tonic-clonic seizures increased to 55.25 ± 6.09 and 62 ± 4.97 s, respectively (*P* < 0.05 vs. saline group), while duration time of tonic-clonic seizures reduced to 35.75 ± 3.59 s. In Vo.Cr (300 mg/Kg) treated group, onset times of myoclonic jerks and tonic-clonic seizures increased to 75 ± 1.58 and 79.25 ± 2.56 s, respectively (*P* < 0.001 vs. saline group), while duration time of tonic-clonic seizures reduced to 30.25 ± 2.32 s (*P* < 0.05 vs. saline group). In Vo.Cr (500 mg/Kg) treated group, onset times of myoclonic jerks and tonic-clonic seizures increased to 87.25 ± 4.03 and 118.25 ± 3.79 s, respectively, while duration time of tonic-clonic seizures reduced to 16.25 ± 2.39 s (*P* < 0.001 vs. saline group). In diazepam (1 mg/Kg) treated group, onset times of myoclonic jerks and tonic-clonic seizures increased to 90.5 ± 2.53 and 130 ± 1.87 s, respectively, while duration time of tonic-clonic seizures reduced to 12.5 ± 2.33 s (*P* < 0.001 vs. saline group). The saline treated group animals showed 100% mortality and immediately died. Vo.Cr at 100 mg/Kg and 300–500 mg/Kg doses reduced PTZ-induced seizures mortality rate to 75 and 0% (*P* < 0.05, *P* < 0.001 vs. saline group) respectively, while diazepam at test dose of 1 mg/Kg, reduced mortality to 0% (*P* < 0.001 vs. saline group) as shown in **Table 1**.

**Table 1:** Effect of *Verbena officinalis* crude extract (Vo.Cr) and diazepam on pentylenetetrazole (PTZ)-induced seizures mortality in mice.

Groups	% Mortality
Saline (10 mL/Kg) + PTZ (90 mg/Kg)	100.00
Vo.Cr (100 mg/Kg) + PTZ (90 mg/Kg)	75.00^∗^
Vo.Cr (300 mg/Kg) + PTZ (90 mg/Kg)	0.00^∗∗∗^
Vo.Cr (500 mg/Kg) + PTZ (90 mg/Kg)	0.00^∗∗∗^
Diazepam (1 mg/Kg) + PTZ (90 mg/Kg)	0.00^∗∗∗^


*Mortality = number of mice died after convulsion/total number of mice used where % mortality = (number of mice died after convulsion/total number of mice used) × 100. Data expressed as mean ± SEM, *n* = 4. ^∗^*P* < 0.05, ^∗∗∗^*P* < 0.001 vs. saline group, one-way ANOVA with *post-hoc* Tukey test.*

### Effect on Time Spent in Open and Closed Arms

**FIGURE 2:**
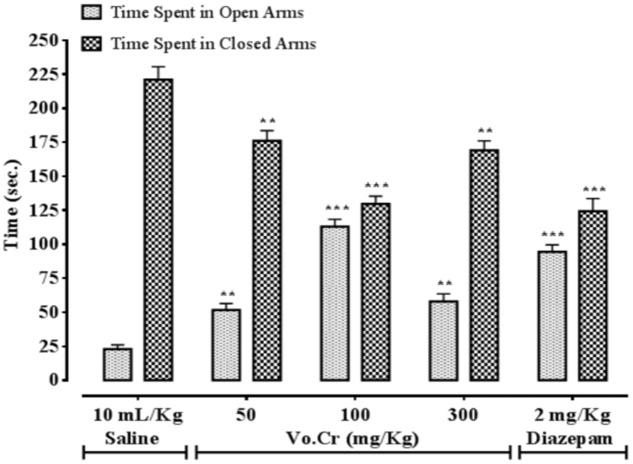
**Bar-graph showing effects of *Verbena officinalis* crude extract (Vo.Cr) and diazepam on time spent by mice in open and closed arms in elevated plus maze model.** Data expressed as mean ± SEM, *n* = 4. ^∗∗^*P* < 0.01, ^∗∗∗^*P* < 0.001 vs. saline group, one-way ANOVA with *post hoc* Tukey test.

Vo.Cr at 50–300 mg/Kg increased time spent by animals in open arms, while decreased time spent in closed arms (**Figure 2**). In control saline group, time spent in open and closed arms were 23 ± 3.11 and 221 ± 9.57 s, respectively. In Vo.Cr (50 mg/Kg) treated group, time spent in open arms increased to 51.75 ± 4.72 s while time spent in closed arms reduced to 176 ± 7.52 s (*P* < 0.01 vs. saline group). In Vo.Cr (100 mg/Kg) treated group, time spent in open arms increased to 113 ± 5.31 s while time spent in closed arms reduced to 129.75 ± 5.59 s (*P* < 0.001 vs. saline group). In Vo.Cr (300 mg/Kg) treated group, time spent in open arms was 58 ± 5.58 s while time spent in closed arms was 169 ± 7.04 s (*P* < 0.01 vs. saline group). In diazepam (2 mg/Kg) treated group, time spent in open arms was increased to 94.5 ± 5.06 s while time spent in closed arms reduced to 124.25 ± 9.41 s (*P* < 0.001 vs. saline group).

### Effect on Number of Entries in Open and Closed Arms

**FIGURE 3:**
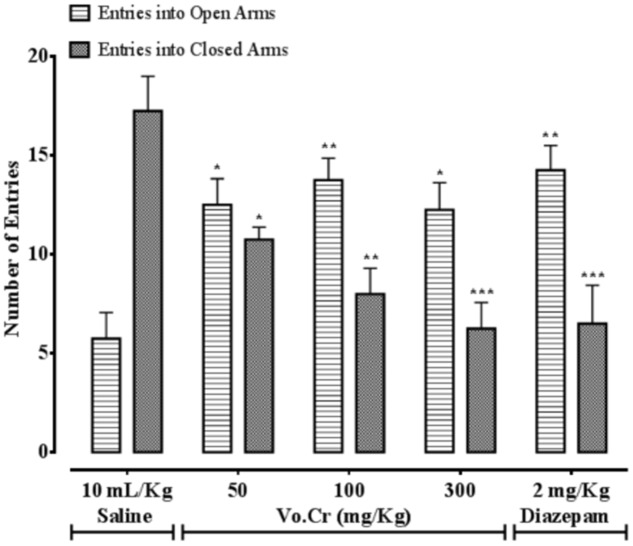
**Bar-graph showing effects of *Verbena officinalis* crude extract (Vo.Cr) and diazepam on numbers of mice entries in open and closed arms in elevated plus maze model.** Data expressed as mean ± SEM, *n* = 4. ^∗^*P* < 0.05, ^∗∗^*P* < 0.01, ^∗∗∗^*P* < 0.001 vs. saline group, one-way ANOVA with *post hoc* Tukey test.

Vo.Cr at 50–300 mg/Kg increased number of animals’ entries into open arms, while decreased number of entries into closed arms (**Figure 3**). In control saline group, numbers of entries into open and closed arms were 5.75 ± 1.32 and 17.25 ± 1.75, respectively. In Vo.Cr (50 mg/Kg) treated group, number of entries into open arms increased to 12.5 ± 1.32, while number of entries in closed arms reduced to 10.75 ± 0.63 (*P* < 0.05 vs. saline group). In Vo.Cr (100 mg/Kg) treated group, number of entries into open arms increased to 13.75 ± 1.11, while number of entries in closed arms reduced to 8.0 ± 1.29 (*P* < 0.01 vs. saline group). In Vo.Cr (300 mg/Kg) treated group, number of entries into open arms were 12.25 ± 1.38 (*P* < 0.05 vs. saline group), while number of entries in closed arms reduced to 6.25 ± 1.32 (*P* < 0.001 vs. saline group). In diazepam (2 mg/Kg) treated group, number of entries into open arms increased to 14.25 ± 1.25 (*P* < 0.01 vs. saline group), while number of entries in closed arms reduced to 6.5 ± 1.94 (*P* < 0.001 vs. saline group).

### Effect on Time Spent in Light and Dark Compartments

**FIGURE 4:**
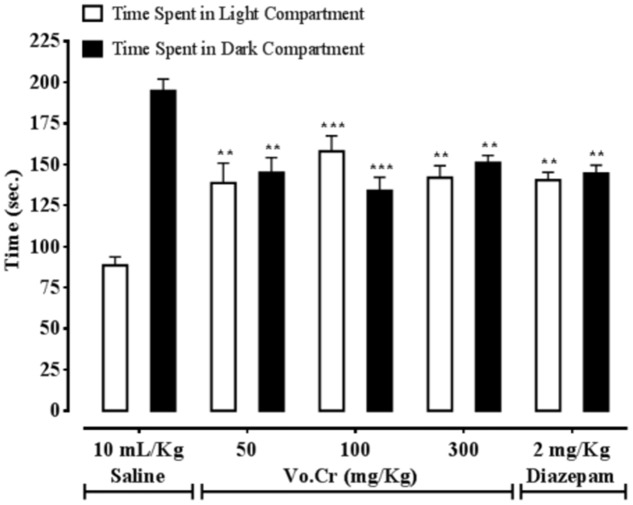
**Bar-graph showing effects of *Verbena officinalis* crude extract (Vo.Cr) and diazepam on time spent by mice in light and dark compartments in light–dark box model.** Data expressed as mean ± SEM, *n* = 4. ^∗∗^*P* < 0.01, ^∗∗∗^*P* < 0.001 vs. saline group, one-way ANOVA with *post hoc* Tukey test.

Vo.Cr at 50–300 mg/Kg increased time spent by animals in light compartment, while decreased time spent in dark compartment (**Figure 4**). In control saline group, times spent in light and dark compartments were 88.5 ± 5.25 and 194.75 ± 7.25 s, respectively. In Vo.Cr (50 mg/Kg) treated group, time spent in light compartment increased to 138.75 ± 12.13 s, while time spent in dark compartment reduced to 145 ± 9.25 s (*P* < 0.01 vs. saline group). In Vo.Cr (100 mg/Kg) treated group, time spent in light compartment increased to 158 ± 9.46 s, while time spent in dark compartment reduced to 134 ± 8.27 s (*P* < 0.001 vs. saline group). In Vo.Cr (300 mg/Kg) treated group, time spent in light compartment was 142 ± 7.22 s, while time spent in dark compartment was 151 ± 4.53 s (*P* < 0.01 vs. saline group). In diazepam (2 mg/Kg) treated group, time spent in light compartment was 140.5 ± 4.74 s, while time spent in dark compartment was 144.5 ± 5.12 s (*P* < 0.01 vs. saline group).

### Effect on Numbers of Ambulations, Rearings, and Central Squares Crossings

**FIGURE 5:**
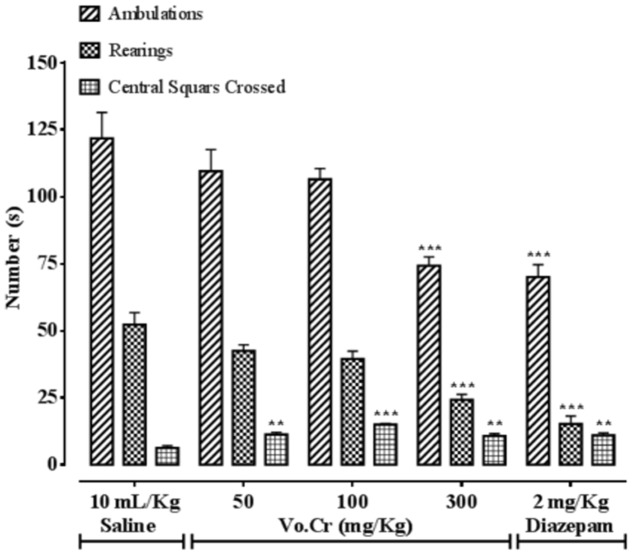
**Bar-graph showing effects of *Verbena officinalis* crude extract (Vo.Cr) and diazepam on numbers of mice ambulations, rearings, and central squares crossings in open field model.** Data expressed as mean ± SEM, *n* = 4. ^∗∗^*P* < 0.01, ^∗∗∗^*P* < 0.001 vs. saline group, one-way ANOVA with *post hoc* Tukey test.

Vo.Cr at highest tested dose of 300 mg/Kg, significantly decreased numbers of animals’ ambulations and rearings frequencies, while increased the number of central squares crossings at all tested doses (50–300 mg/Kg) compared to control saline group, as presented in **Figure 5**. In saline group, numbers of ambulations, rearings, and central squares crossings were 121.75 ± 9.71, 52.25 ± 4.52, and 6.25 ± 0.85, respectively. In Vo.Cr (50 mg/Kg) treated group, numbers of ambulations and rearings were 109.5 ± 8.06 and 42.5 ± 2.22 (*P* > 0.05 vs. saline group) respectively, while number of central squares crossings increased to 11.25 ± 0.85 (*P* < 0.01 vs. saline group). In Vo.Cr (100 mg/Kg) treated group, numbers of ambulations and rearings were 106.5 ± 4.03 and 39.5 ± 2.9 (*P* > 0.05 vs. saline group) respectively, while number of central squares crossings increased to 15 ± 0.41 (*P* < 0.001 vs. saline group). In Vo.Cr (300 mg/Kg) treated group, numbers of ambulations and rearings reduced to 74.25 ± 3.30 and 24.25 ± 1.97 respectively (*P* < 0.001 vs. saline group), while number of central squares crossings were 10.75 ± 0.85 (*P* < 0.01 vs. saline group). In diazepam (2 mg/Kg) treated group, numbers of ambulations and rearings decreased to 70 ± 4.66 and 15.25 ± 2.96 respectively (*P* < 0.001 vs. saline group), while number of central squares crossings increased to 11 ± 0.91 (*P* < 0.01 vs. saline group).

### Effect on Thiopental-Induced Sleeping Time

**FIGURE 6:**
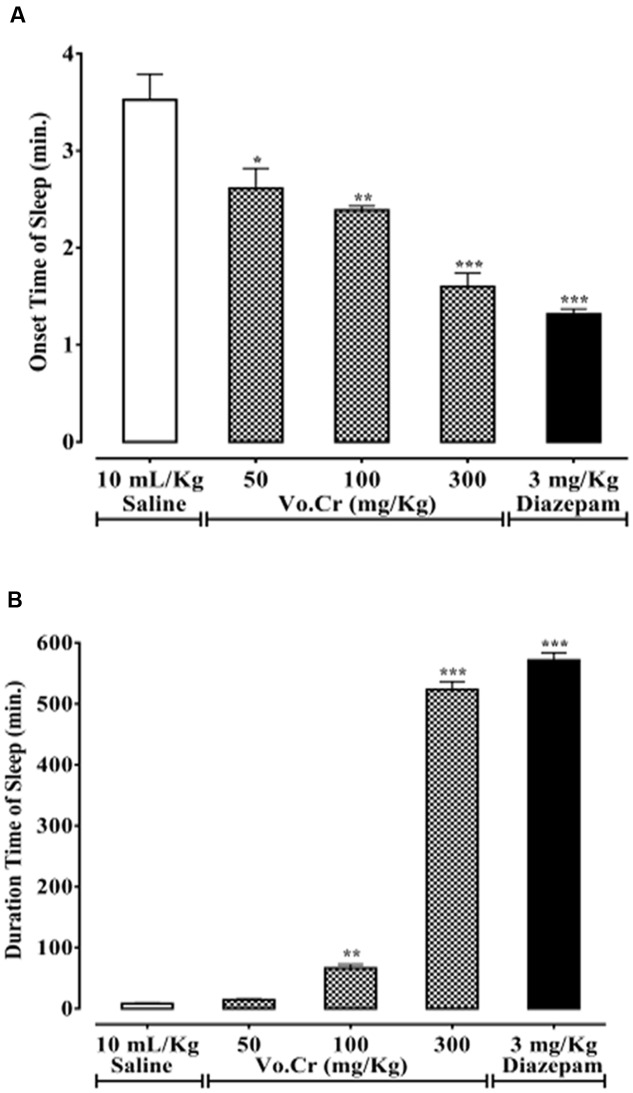
**Bar-graph showing effects of *Verbena officinalis* crude extract (Vo.Cr) and diazepam on** **(A)** onset time of sleep and **(B)** duration of sleep in thiopental-induced sleeping test in mice. Data expressed as mean ± SEM, *n* = 4. ^∗^*P* < 0.05, ^∗∗^*P* < 0.01, ^∗∗∗^*P* < 0.001 vs. saline group, one-way ANOVA with *post hoc* Tukey test.

Vo.Cr dose-dependently (50–300 mg/Kg) decreased animals’ onset time of sleep, while increased sleep duration (**Figures 6A,B**). In control saline group, onset and duration of sleep times were 3.53 ± 0.27 and 8.25 ± 1.16 min, respectively. In Vo.Cr (50 mg/Kg) treated group, onset time of sleep decreased to 2.61 ± 0.21 min (*P* < 0.05 vs. saline group), while duration of sleep time was increased to 14.50 ± 1.57 min. In Vo.Cr (100 mg/Kg) treated group, onset time of sleep was decreased to 2.39 ± 0.04 min, while duration of sleep time was increased to 66.45 ± 6.13 min (*P* < 0.01 vs. saline group). In Vo.Cr (300 mg/Kg) treated group, onset time of sleep was further decreased to 1.6 ± 0.14 min, while duration of sleep time was increased to 523.65 ± 13.03 min (*P* < 0.001 vs. saline group). In diazepam (3 mg/Kg) treated group, onset time of sleep was 1.32 ± 0.05 min, while duration of sleep time was 571.8 ± 12.17 min (*P* < 0.001 vs. saline group).

### Acute Toxicity

Vo.Cr at higher doses of 3 and 5 g/Kg, i.p. did not caused any mortality, but only decreased locomotor activity of mice showing sedative effect.

## Discussion

We employed different pharmacological techniques, to investigate anticonvulsant, anxiolytic and sedative effects of *Verbena officinalis*. PTZ-induced seizure is commonly used animal model for screening of anti-seizure drugs (Loscher and Schmidt, 1988; Okoye et al., 2013). Vo.Cr caused marked delay in onset time of both myoclonic jerks and tonic-clonic seizures, as well as decreased duration of tonic-clonic seizures and animals’ mortality, demonstrating its anti-epileptic effect. PTZ evokes convulsions via inhibition of GABAergeic neurotransmissions by interfering with GABA_A_ receptors (Ramanjaneyulu and Ticku, 1984). Dr. Thomas B. Turnbaugh in his professional experience observed verbenin, an iridoid glycoside, obtained from *Verbena hastate* more beneficial than bromide for epilepsy treatment. He favored verbenin that it has no injurious effects and its use makes the patient more cheerful and alert instead of bromide which makes the patient stupid and dull (French, 1908). Verbenin is also the main constituent of *Verbena officinalis* (Duke, 1992) which might be responsible for its anticonvulsant activity. *Verbena officinalis* is known to contain a constituent, 4-(1-methylethyl)-benzylic alcohol safranal (Gharachorloo and Amouheidari, 2016) and safranal from *Crocus sativus* plant has been reported for its anti-seizure action by virtue of GABA_A_ receptors stimulation (Hosseinzadeh and Talebzadeh, 2005; Hosseinzadeh and Sadeghnia, 2007), suggesting that anticonvulsant effect of *Verbena officinalis* might possibly occurred through activation of GABA_A_ receptors by safranal. EPM and LDB models are favorable for testing of GABA_A_-receptors linked anxiolytic drugs (Emamghoreishi et al., 2005; Mesfin et al., 2014). Agents, which increase animals’ time spent and number of entries into open arms and/or reducing these phenomenon in closed arms in EPM, also increase animals’ time spent in light compartment and/or reducing these phenomenon in dark compartment in LDB, are considered to possess anxiolytic effects (Hellion-Ibarrola et al., 2006). Vo.Cr significantly increased time spent and number of entries into open arms, while deceased time spent and number of entries into closed arms, as well as increased time spent in light compartment, while deceased time spent in dark compartment, like that caused by diazepam, a standard benzodiazepine anxiolytic medicine (Griebel et al., 1998). At the 100 mg/Kg dose, Vo.Cr showed maximum anxiolytic effect, but at next highest tested dose (300 mg/Kg), its effect was reduced, because of decreased exploratory activities, possibly be due to co-existent sedative component of the plant. *Verbena officinalis* has been reported for presence of flavonoids (apigenin, luteolin, quercetion, kaempferol) and tannins (Mengiste et al., 2014; Edwards et al., 2015), which are well-known for their anxiolytic and central nervous system depressant (sedative) activities (Adeyemi et al., 2006; Coleta et al., 2008; Aguirre-Hernandez et al., 2010). Several flavonoids bind to the benzodiazepine site on GABA_A_-receptors to provoke anti-seizure, anti-anxiety and sedative effects (Jager and Saaby, 2011), evidencing that observed actions of *Verbena officinalis* mediated possibly through activation of GABA_A_-receptors pathway. However, involvement of other contributing mechanism(s) cannot be ignored. In open field test principle, increased number of ambulations, rearings and central squares crossings reveal anxiolytic activity, while reduction in locomotion showed sedative effect. Vo.Cr at lower doses of 50–100 mg/Kg did not produced statistically any prominent difference in ambulation and rearing frequencies, compared to saline group (*P* > 0.05), but both ambulation and rearing frequencies were significantly reduced at highest dose of 300 mg/Kg (*P* < 0.001 vs. saline group). The decreased locomotor activity at higher dose of Vo.Cr might be due to sedative effect of the plant. Vo.Cr, at all tested doses (50–300 mg/Kg) significantly increased numbers of central squares crossings, showing its anxiolytic activity. Diazepam, a reference drug also reduced both ambulation and rearing frequencies, because of its sedative property, while increased the number of central squares crossings, due to owning anxiolytic profile (Ramos, 2008). Benzodiazepines are well-established for anxiolytic effect at lower doses and sedative, muscle relaxant and anti seizure effects at higher doses (Melo et al., 2006). Substances that have sedative effect either decrease onset time of sleep and/or prolong duration of sleep (Raquibul-Hasan et al., 2009). In thiopental-induced sleeping assay, Vo.Cr in dose-dependent manner decreased onset time of sleep, while increased duration of sleep, indicating its sedative activity, further confirming that the decreased locomotor activity of mice in EPM, light–dark and open field tests was due to sedative effect of the plant test extract. Flavonoids like scutellarein and phenolic acid derivatives like verbascoside have been reported for their sedative effects but they have low affinity for benzodiazepine site on GABA_A_ receptor. The authors suggest that they could induce CNS activities through different mechanism like inhibition of the *N*-methyl-D-aspartate or serotonin receptors and the type of sugar linkage with the aglycone should be an important factor for their sedative activities (De Santana Julião et al., 2010). Scutellarein and verbascoside are the main constituents of *Verbena officinalis* (Rehecho et al., 2011), so sedative effect of *Verbena officinalis* may be due to these constituents possibly through GABA pathways by acting on sites other than benzodiazepine ones or through pathways other than GABAergic system.

## Conclusion

The present study reveals that *Verbena officinalis* exhibits anticonvulsant, anxiolytic and sedative effects, which validates its folk use in neurological disorders and a step forward toward exploration of evidence-based alternative medicines. Further in-depth advance molecular studies are warranted to elucidate pharmacodynamics basis of the pharmacological actions.

## Author Contributions

AK performed this research work under the supervision of A-uK and co-supervision TA.

## Conflict of Interest Statement

The authors declare that the research was conducted in the absence of any commercial or financial relationships that could be construed as a potential conflict of interest.
